# Site of clear corneal incision in cataract surgery and its effects on surgically induced astigmatism

**DOI:** 10.1038/s41598-020-60985-5

**Published:** 2020-03-03

**Authors:** Junjie Piao, Choun-Ki Joo

**Affiliations:** 1 Department of Ophthalmology, Peking Union Medical College Hospital, Chinese Academy of Medical Sciences & Peking Union Medical College, Beijing, China; 2 Catholic Institute for Visual Science, College of Medicine, The Catholic University of Korea, Seoul, South Korea; 3 Department of Ophthalmology, Catholic Institute for Visual Science, Seoul St. Mary’s Hospital, College of Medicine, The Catholic University of Korea, Seoul, South Korea

**Keywords:** Corneal diseases, Lens diseases, Refractive errors

## Abstract

Clear corneal incision (CCI) is a commonly used surgical approach in cataract surgery. In this prospective study, we evaluated the effect of CCI site on surgically induced astigmatism (SIA) and other postoperative astigmatic changes. CCIs were constructed based on the steep meridian of the total corneal refractive power in the 4.0-mm-zone (TCRP_4.0_), and patients were divided into four groups: temporal, superotemporal, superonasal, and superior according to the site of the incision. TCRP_4.0_ analysis demonstrated a statistically significant reduction of astigmatism with superior incisions (P < 0.001), and the combined mean polar values for SIA changed significantly in the temporal (Hotelling T^2^ = 1.977), superotemporal (Hotelling T^2^ = 0.544), superonsal (Hotelling T^2^ = 1.066), and superior incision groups (Hotelling T^2^ = 1.134) (all P < 0.001). The posterior axis alignment should be considered in cataract surgery with CCI, and the SIA is affected by axis rotation, and incision orientation.

## Introduction

For an increasing number of younger patients diagnosed with cataract or presbyopia, refractive surgery is becoming an option to correct refractive errors. It not only improves visual acuity, but also vision quality. Since 1990, sutureless clear corneal incisions (CCI) have become the standard option in cataract surgery to minimize postoperative astigmatism and promote rapid visual rehabilitation^1,2^. However, corneal surgically-induced astigmatism (SIA) still occurs, and studies have reported that the severity of SIA depends on the length, width, and site of the incision^3–6^.

Today, precise estimation of corneal power and astigmatism is critical for anterior segment refractive surgery. Until now, corneal power and astigmatism have been measured using only the anterior corneal curvature, which is measured by methods such as manual keratometry, auto-keratometry, and simulated keratometry (Sim-K). All of these neglect the contribution of posterior corneal astigmatism. Therefore, only a few studies have described posterior corneal shape changes after cataract surgery^6–10^. The Pentacam is a diagnostic tool that uses a slit illumination system and works according to the Scheimpflug principle; it can directly measure both anterior and posterior corneal curvatures^11^.

Shajari *et al*.^12^ demonstrated that the parameters for differentiating keratoconus from healthy eyes included pachymetry, corneal curvature, and anterior and posterior corneal axis alignments. To date, the most accurate method for determining corneal astigmatism axis alignment remains unclear. However, posterior axis alignment is most likely vertical. Mean posterior corneal astigmatism values ranging from −0.26 to −0.78 D have been reported^12–16^. Despite numerous studies, there is still significant confusion regarding the best method to evaluate SIA of the cornea^17–19^. Abulafia *et al*. recently provided a new definition for SIA of the cornea (SIA_Cornea_)^20^.

The aim of the current prospective study was to determine the effects of the site of CCI on the total corneal refractive power (TCRP) at 4.0-mm zone (TCRP_4.0_) steep axis after cataract surgery.

## Results

In post-hoc comparison analysis of deviation of means between the groups, SIA_Cornea_ differed significantly with age (P = 0.001). There were no statistically significant differences in any preoperative demographic data between the four groups (Table 1).

**Table 1:** Preoperative demographic data.

Parameter	Incision Group				*P* value
	Temporal	Superotemporal	Superonasal	Superior	
Eyes (n)	49	49	61	53	—
Male/Female (n)	8/38	12/36	22/34	15/33	—
Mean age (y) ± SD	70.14 ± 9.15	65.14 ± 12.17	67.49 ± 11.47	61.40 ± 11.18	0.001
TCRP_4.0_ K_mean_ (D)	43.42 ± 4.57	44.05 ± 2.04	44.04 ± 1.38	44.06 ± 1.49	0.687
TCRP_3.0_ K_mean_ (D)	43.73 ± 1.21	44.32 ± 2.09	44.23 ± 1.21	44.33 ± 1.41	0.614
Sim-K_Ant._ K_mean_ (D)	43.00 ± 4.56	43.63 ± 2.20	43.53 ± 1.16	42.94 ± 5.02	0.310
SIA_Cornea_ (D)	0.27 ± 0.19	0.34 ± 0.35	0.38 ± 0.35	0.40 ± 0.37	0.310
IOL Power (D)	19.39 ± 4.38	19.32 ± 4.36	19.56 ± 3.88	18.58 ± 4.14	0.623
ECD (mm^2^)	2679.71 ± 283.60	2728.06 ± 280.74	2664.41 ± 315.80	2696.42 ± 273.07	0.705

y = year; SD = standard deviation; TCRP_4_ = total corneal refractive power at 4.0-mm-zone; TCRP_3_ = total corneal refractive power at 3.0-mm-zone; Sim-K = simulated karatometry; Ant. = anterior corneal curvature; K_mean_ = average of keratometry; D = diopters; IOL = intraocular lens; ECD = endothelium cell density.

There were statistically significant changes in TCRP_4.0_ polar values in each group (all P < 0.001; Hotelling T^2^ = 1.977 in the temporal; Hotelling T^2^ = 0.544 in the superotemporal; Hotelling T^2^ = 1.066 in the superonasal; Hotelling T^2^ = 1.134 in the superior) (Table 2). TCRP_3.0_ polar values were statistically significant changed in each group (all P < 0.001; Hotelling T^2^ = 1.777 in the temporal; Hotelling T^2^ = 0.698 in the superotemporal; Hotelling T^2^ = 1.203 in the superonasal; Hotelling T^2^ = 1.004 in the superior) (Table 3). However, Sim-K_Ant._ polar values were also statistically significant changed in all incision groups (all P < 0.001; Hotelling T^2^ = 1.713 in the temporal; Hotelling T^2^ = 0.680 in the superotemporal; Hotelling T^2^ = 1.464 in the superonasal; Hotelling T^2^ = 1.660 in the superior). In univariate analysis the AKP(+0) decreased significantly in all incision groups, and AKP(+45)—which is a representation of torsional force twisting in the astigmatic direction—was not significant after 2 months (Table 4).

**Table 2:** Polar value analysis of TCRP4.0.

Group	Mean Keratometry (D) ± SD						*P* Value^a^	*P* Value^b^	
	Preoperative		Postoperative		Change				
	AKP(+0)	AKP(+45)	AKP(+0)	AKP(+45)	△AKP(+0)	△AKP(+45)		△AKP(+0)	△AKP(+45)
Temporal	0.87 ± 0.45	0	−0.09 ± 0.62	−0.04 ± 0.64	−0.96 ± 0.76	−0.04 ± 0.64	<0.001	<0.001	0.629
Superotemporal	0.99 ± 1.02	0	−0.07 ± 1.05	−0.02 ± 0.74	−1.06 ± 1.79	−0.02 ± 0.74	<0.001	<0.001	0.887
Superonasal	0.90 ± 0.62	0	0.16 ± 0.81	−0.10 ± 0.67	−0.73 ± 0.96	−0.10 ± 0.67	<0.001	<0.001	0.234
Superior	1.12 ± 0.75	0	0.24 ± 0.88	−0.03 ± 0.74	−0.87 ± 1.05	−0.03 ± 0.74	<0.001	<0.001	0.774

D = diopters; SD = standard deviation; △ = change; AKP = astigmatic polar value.

^a^Hotelling trace between preoperative and postoperative keratometry.

^b^Univariate analysis for △AKP(+0) and △AKP(+45).

**Table 3:** Polar value analysis of TCRP_3.0_.

Group	Mean Keratometry (D) ± SD						*P* Value^a^	*P* Value^b^	
	Preoperative		Postoperative		Change				
	AKP(+0)	AKP(+45)	AKP(+0)	AKP(+45)	△AKP(+0)	△AKP(+45)		△AKP(+0)	△AKP(+45)
Temporal	0.88 ± 0.48	0	−0.50 ± 0.57	0.04 ± 0.73	−1.37 ± 0.79	0.04 ± 0.73	<0.001	<0.001	0.697
Superotemporal	0.73 ± 0.63	0	−0.29 ± 0.58	−0.04 ± 0.70	−1.02 ± 1.01	−0.04 ± 0.70	<0.001	<0.001	0.671
Superonasal	0.80 ± 0.53	0	−0.36 ± 0.69	0.06 ± 0.77	−1.16 ± 0.87	0.06 ± 0.77	<0.001	<0.001	0.553
Superior	0.89 ± 0.63	0	−0.31 ± 0.69	−0.08 ± 0.72	−1.20 ± 1.10	−0.08 ± 0.72	<0.001	<0.001	0.449

D = diopters; SD = standard deviation; △ = change; AKP = astigmatic polar value.

^a^Hotelling trace between preoperative and postoperative keratometry.

^b^Univariate analysis for △AKP(+0) and △AKP(+45).

**Table 4:** Polar value analysis of Sim-K_Ant._.

Group	Mean Keratometry (D) ± SD						*P* Value^a^	*P* Value^b^	
	Preoperative		Postoperative		Change				
	AKP(+0)	AKP(+45)	AKP(+0)	AKP(+45)	△AKP(+0)	△AKP(+45)		△AKP(+0)	△AKP(+45)
Temporal	0.76 ± 0.41	0	−0.04 ± 0.47	−0.03 ± 0.57	−0.80 ± 0.61	−0.03 ± 0.57	<0.001	<0.001	0.698
Superotemporal	0.94 ± 0.83	0	0.04 ± 0.59	0.10 ± 0.83	−0.90 ± 1.05	0.10 ± 0.83	<0.001	<0.001	0.382
Superonasal	0.81 ± 0.48	0	0.05 ± 0.63	0.03 ± 0.66	−0.76 ± 0.79	0.03 ± 0.66	<0.001	<0.001	0.697
Superior	1.10 ± 0.62	0	−0.01 ± 0.95	−0.13 ± 0.69	−1.11 ± 1.08	−0.13 ± 0.69	<0.001	<0.001	0.159

D = diopters; SD = standard deviation; △ = change; AKP = astigmatic polar value.

^a^Hotelling trace between preoperative and postoperative keratometry.

^b^Univariate analysis for △AKP(+0) and △AKP(+45).

The changes of keratometric astigmatism in TCRP_4.0_, TCRP_3.0_, and Sim-K_Ant._ were shown in Table 5. There was statistically significant reduction of astigmatism in TCRP_4.0_ on the site of superior direction (P < 0.001), and significant reduction of astigmatism in Sim-K_Ant._ on the site of superotemporal, superonasal, and superior directions. There were no statistically significant differences in K_mean_ between preoperative and postoperative in TCRP_4.0_, TCRP_3.0_, and Sim-K_Ant._ (all P > 0.05, Table 6).

**Table 5:** Preoperative and postoperative keratometric astigmatism changes.

Parameter	Incision Group				*P* Value*
	Temporal	Superotemporal	Superonasal	Superior	
**TCRP**_**4.0**_					
Pre-op Ast. (D)	0.87 ± 0.45	0.99 ± 1.02	0.90 ± 0.62	1.12 ± 0.75	0.177
Post-op Ast. (D)	0.79 ± 0.47	0.94 ± 1.00	0.89 ± 0.59	0.86 ± 0.73	0.145
*P* value	0.055	0.332	0.621	<0.001	
**TCRP**_3.0_					
Pre-op Ast. (D)	0.88 ± 0.48	0.73 ± 0.63	0.80 ± 0.53	0.89 ± 0.63	0.265
Post-op Ast. (D)	0.79 ± 0.49	0.74 ± 0.48	0.91 ± 0.57	0.80 ± 0.77	0.871
*P* value	0.281	0.801	0.212	0.110	
**Sim-K**_**Ant.**_					
Pre-op Ast. (D)	0.76 ± 0.41	0.94 ± 0.83	0.81 ± 0.48	1.10 ± 0.62	0.004
Post-op Ast. (D)	0.60 ± 0.41	0.79 ± 0.68	0.71 ± 0.48	0.92 ± 0.71	0.073
*P* value	0.095	0.004	0.042	<0.001	

TCRP_4.0_ = total corneal refractive power at 4.0-mm-zone; TCRP_3.0_ = total corneal refractive power at 3.0-mm-zone; Pre-op Ast. = Preoperative astigmatism; Post-op Ast. = Postoperative astigmatism; D = diopters; Sim-K = simulated keratometry; Ant. = anterior corneal curvature.

**Table 6:** Preoperative and postoperative K_mean_ changes in TCRP_4.0_, TCRP_3.0_, Sim-K_Ant._, and Sim-K_Post._.

Parameter	Incision Group				*P* Value
	Temporal	Superotemporal	Superonasal	Superior	
**TCRP**_**4.0**_					
Pre-op K_mean_ (D)	43.63 ± 1.42	44.05 ± 2.04	44.04 ± 1.38	44.06 ± 1.49	0.472
Post-op K_mean_ (D)	43.63 ± 1.40	43.99 ± 2.08	44.07 ± 1.38	44.03 ± 1.44	0.511
**TCRP**_**3.0**_					
Pre-op K_mean_ (D)	43.21 ± 1.43	43.63 ± 2.20	43.53 ± 1.16	43.64 ± 1.45	0.482
Post-op K_mean_ (D)	43.10 ± 1.36	43.59 ± 2.29	43.52 ± 1.25	43.62 ± 1.39	0.339
**Sim-K**_**Ant.**_					
Pre-op K_mean_ (D)	43.99 ± 1.33	44.32 ± 2.09	44.23 ± 1.21	44.33 ± 1.41	0.663
Post-op K_mean_ (D)	43.93 ± 1.33	44.31 ± 2.22	44.22 ± 1.25	44.33 ± 1.36	0.549
**Sim-K**_**Post.**_					
Pre-op K_mean_ (D)	−6.41 ± 0.23	−6.46 ± 0.43	−6.42 ± 0.21	−6.41 ± 0.24	0.746
Post-op K_mean_ (D)	−6.43 ± 0.24	−6.49 ± 0.44	−6.43 ± 0.24	−6.44 ± 0.25	0.730

TCRP_4.0_ = total corneal refractive power at 4.0-mm-zone; TCRP_3.0_ = total corneal refractive power at 3.0-mm-zone; Sim-K = simulated keratometry; Ant. = Anterior corneal curvature; Post. = Posterior corneal curvature; D = diopters; Pre-op K_mean_ = preoperative average of keratometry; Post-op K_mean_ = postoperative average of keratometry.

**Figure 1:**
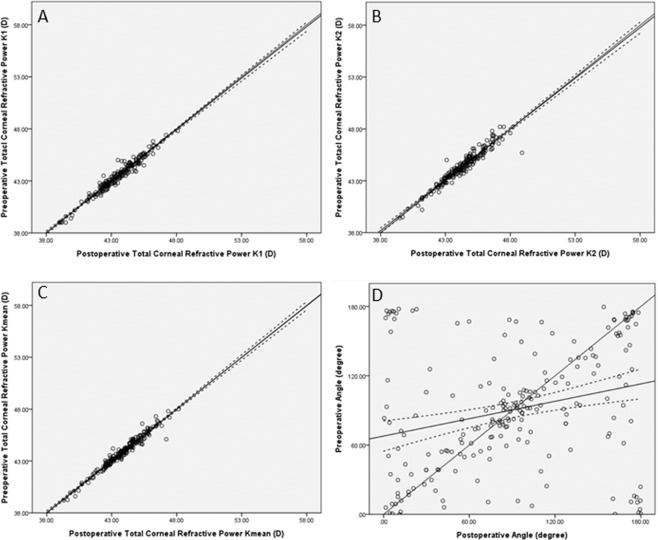
Equivalency plot of the preoperative keratometry value and angle of astigmatism on the y-axis versus the postoperative keratometry value and angle of astigmatism on the x-axis: (**A**). K_1_ (flattest keratometry); (**B**). K_2_ (steepest keratometry); (**C**). K_mean_ (average of keratometry); and (**D**). Angle of axis.

Overall, 95.8% of the cases were within the 95% confidence intervals of different values of flattest keratometry (K_1_), with R-square (R^2^) = 0.95, adjusted R^2^ = 0.95, and standard error = 0.34 D. For steepest keratometry (K_2_), 97.6% of the cases were within the 95% confidence intervals of different values of K_2_, with R^2^ = 0.93, adjusted R^2^ = 0.93, and standard error = 0.44 D. For the average of keratometry (K_mean_), 95.3% of the cases were within the 95% confidence intervals of different values of K_mean_, with R^2^ = 0.96, adjusted R^2^ = 0.96 and standard error = 0.30 D. For the angle of astigmatism, 91.5% of the cases were within the 95% confidence intervals of different values, with R^2^ = 0.05, adjusted R^2^ = 0.05, and standard error = 50.6 degrees (Fig. 1).

**Figure 2:**
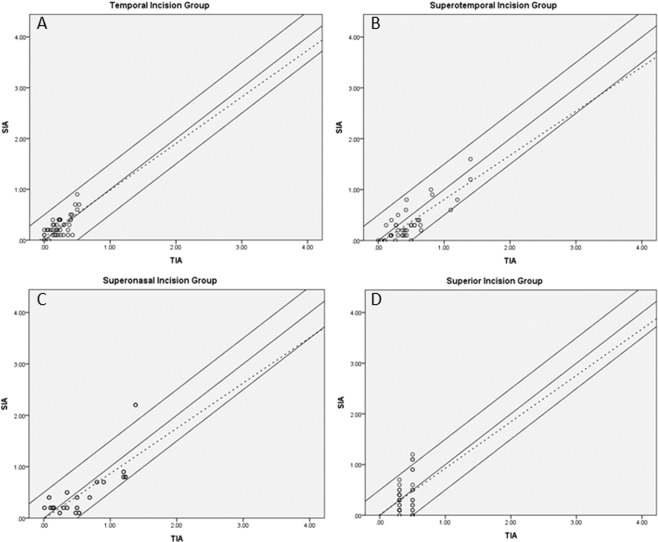
Effect of TCRP_4.0_ (total corneal refractive power at 4 mm) steep meridian incision. Individual corneal astigmatism correction (Alpins) in: (**A**). Temporal group; (**B**). Superotemporal group; (**C**). Superonasal group; and (**D**). Superior group.

The difference of correction index between the magnitude of Predicted SIA_Cornea_ and SIA_Cornea_ was less than 0.50 D in 43 of 49 eyes (87.8%) in the temporal group, 39 of 49 eyes (79.6%) in the superotemporal group, 46 of 61 eyes (75.4%) in the superonasal group, and 40 of 53 eyes (75.5%) in the superior group (Fig. 2).

## Discussion

Multiple studies have investigated the effects of posterior surface astigmatism and curvature in refractive surgery, especially in cases of keratoconus. Implantation of premium IOLs, particularly multifocal and toric IOLs in cataract surgery, requires reliable prediction of the refractive results. Given the controllable nature of postoperative corneal astigmatism and visual quality after operations^21–25^, the present study aimed to analyze the effects of the site of CCI on TCRP_4.0_ steep meridians in cataractous eyes.

Keratometry has traditionally been the favored method for evaluating astigmatic changes; however, it cannot be used to evaluate the peripheral cornea or irregularities of the central cornea^26,27^. In order to conduct a thorough, detailed, and objective evaluation of corneal tomography and topography, computer-assisted video keratography (CVK) is widely used in optometry and ophthalmology^5^. The Pentacam biometric device provides precise values for corneal power and topometric indices, both in normal and in keratoconic eyes^28^. In the current study, Scheimpflug tomography was used to analyze anterior and posterior curvatures based on a ray tracing technique combined with a rotating Scheimpflug camera.

According to a previous study, the percentage of eyes with vertical alignment of the steep meridian decreased with increasing age (from 64% in patients aged 60–69 years to 48.1% in those aged 70–79 years), and the percentage of patients with horizontal alignment increased from 17.5% to 31.8% for the anterior corneal surfaces; however, almost cases of eyes in vertical alignment on the posterior corneal surface^11^. In our study, patients in the 60–69 years age group were more likely to undergo superior incision, while patients in the 70–79 years age group were more likely to undergo temporal incision. In principle, when the steep meridian on the anterior surface is aligned vertically, it creates with-the-rule (WTR) astigmatism; in contrast, vertical alignment on the posterior surface creates against-the-rule (ATR) astigmatism, which has negative power and partially compensates for anterior astigmatism.

In 2012, Rho *et al*.^4^ reported that steep meridian incision in CCI surgery was associated with significantly reduced keratometric astigmatism at the temporal, superotemporal, and superior locations. Astigmatic polar value of net astigmatism (AKP)^19^ analysis was used in that study; the changes in AKP(+0) were −0.26 ± 0.53 D in a temporal group, −0.39 ± 0.45 D in a superotemporal group, and −0.46 ± 0.63 D in a superior group. Moreover, there were significant decreases in astigmatism values in AKP(+0), but no significant changes in AKP(+45). In the analysis of the effects of CCI in each steep meridian axis group in the current study, superior incisions were more effective than the others (P < 0.001). In the present study, the changes in AKP(+0) were −0.96 ± 0.76 D in the temporal group, −1.06 ± 1.79 D in the superotemporal group, −0.73 ± 0.96 D in the superonasal group, and −0.87 ± 1.05 D in the superior group, and all of these changes were statistically significant (TCRP_4.0_). Moreover, the changes of TCRP_3.0_ in AKP(+0) were −1.37 ± 0.79 D, −1.02 ± 1.01 D, −1.16 ± 0.87 D, and −1.20 ± 1.10 D, respectively; the changes of Sim-K_Ant._ in AKP(+0) were −0.80 ± 0.61 D, −0.90 ± 1.05 D, −0.76 ± 0.79 D, and −1.11 ± 1.08 D, respectively. Notably, however, torsional force twisting in the astigmatic direction, represented by AKP(+45) values was not significant after 2 months. These results were similar to the results of previously reported studies involving similar groups^4^, but the changes in SIA_Cornea_ AKP(+0) polar values were greater in our study. Moreover, analysis the reduction of astigmatism, there were statistically significant in Sim-K_Ant._ on the incision site of superotemporal, superonasal, and superior directions, and significant reduction of astigmatism in TCRP_4.0_ was investigated on the incision site of superior direction (all P < 0.05).

In the present study, the Alpins method^29^ was used to investigate the effect of each incision site on SIA_Cornea_. Scatterplots of the astigmatic correction, SIA_Cornea_/Predicted SIA_Cornea_, depicted a greater bias toward unity and zero in each group. The values were widely and more evenly distributed, suggesting a lower potential for intended induced astigmatism. With regard to corneal anatomy, the distance from the cornea to the limbus is shorter in the horizontal direction than the vertical direction^30^. Moreover, as the strength of corneal fibers is much stronger for short distances from the central cornea, it is reasonable to suggest that there are changes in the morphology of the cornea and astigmatism after surgery^31–33^. Hence, the SIA_Cornea_ is different depending on the location of CCI. In current study, we investigated the mean and standard deviation values of SIA_Cornea_ in each location of CCI were 0.27 ± 0.19 D, 0.34 ± 0.35 D, 0.38 ± 0.35 D, and 0.40 ± 0.37 D, respectively (P = 0.310). Slade *et al*.^34^ reported that the image-guided surgical planning system was effective for accurate correction of preexisting astigmatism in cataract surgery, especially when combined with femtosecond laser-assisted cataract surgery. Byun *et al*.^35^ studied similar groups and suggested that anterior keratometry does not accurately reflect total corneal astigmatism. They also reported that the correction index was higher with ATR astigmatism than with WTR astigmatism. Accordingly, the refractive cylinder was higher than the keratometric astigmatism in the ATR group and the oblique group, and lower in eyes with WTR astigmatism. However, the similar correction index was investigated in each group in the present study, and superior outcomes were found in the ATR and WTR groups.

Alió *et al*.^36^ reported that SIA was influenced by the size and location of the incision, and Denoyer *et al*.^37^ proposed that corneal biomechanical properties should be taken into account when making the incision in refractive surgery. In the current study, SIA_Cornea_ was influenced by the steep meridian axis location, irrespective of the incision width. Furthermore, there was a statistically significant difference between preoperative and postoperative K_mean_ (TCRP_4.0_) values (a difference of 0.16 ± 0.30 D), but there were no statistically significant differences in TCRP_3.0_ and Sim-K_Ant._ (a difference of 0.02 ± 0.32 D and 0.04 ± 0.49 D, respectively).

As recommended by Alpins *et al*.^38^, the flattening effect at the incision meridian should be used for toric IOL calculation. In a previous study, Ferreira *et al*.^39^ compared flattening effects after CCI in a femtosecond laser group and a manual group, which involves making the side port at 90 to 110 degrees from the main incision. They reported flattening effects of −0.11 D in the femtosecond laser group and −0.13 D in the manual group, and additionally, with regard to superior oblique incisions, the values were −0.21 D in the femtosecond laser group and −0.34 D in the manual group. Interestingly, in the present study, the flattening effects were −0.04 ± 0.26 D in the temporal group, −0.06 ± 0.27 D in the superotemporal group, 0.03 ± 0.38 D in the superonasal group, and −0.03 ± 0.33 D in the superior group.

Park and Kim^8^ recently reported the effects of CCI on the TCRP_3.0_ steep meridian axis. The procedure they utilized involved the use of a 1-mm side port, 70 degrees to the left of the main incision, including angles of incisions that were 40 degrees larger than those in the groups in the current study, at each location. According to their results, total corneal astigmatism (TCA – 3 mm apex/zone data from the Scheimpflug) was associated with significant changes in AKP(+0) and AKP(+45) 2 months postoperatively. The AKP(+0) means and standard deviations were −0.22 ± 0.20 D in the WTR microcoaxial group and −0.20 ± 0.21 D in the ATR microcoaxial group, and the respective AKP(+45) values were 0.12 ± 0.10 D and 0.11 ± 0.10 D. However, there were no statistically significant changes in keratometric astigmatism. Nevertheless, their study indicated that steep meridian incisions may have a significant torsional effect and that TCA is an effective tool for analysis of corneal astigmatic changes after cataract surgery. However, in our study, the postoperative AKP(+0) of Sim-K_Ant._ were −0.04 ± 0.47 D, 0.04 ± 0.59 D, 0.05 ± 0.63 D, and −0.01 ± 0.95 D, respectively, and it was much lower than the results of the previous study.

The present study has several limitations. It has been reported that incision width and corneal hysteresis influence SIA_Cornea_^37^. Dry eye syndrome remains one of the postoperative symptoms experienced by some patients^40–42^, and is associated with ocular soreness, pain, and a burning sensation, among other things. In an analysis reported by Kasetsuwan *et al*.^40^, only 9.8% of eyes had dry eye symptoms after 3 months following cataract surgery; in the present study, patients with severe dry eyes were excluded. In addition, only Pentacam measurements labeled with the quality specification “OK” were accepted as valid in the current study. These factors could not affect the postoperative evaluation of SIA_Cornea_. In the context of study limitations, notably optimized corneal incision sites would improve ocular aberration results^25–27^. Further studies are required for comparative evaluation of their effects on SIA_Cornea_ and corneal higher-order aberrations (HOAs).

In conclusion, unsutured CCI on the TCRP_4.0_ steep meridian is effective in reducing SIA_Cornea_ changes, and has a slight flattening effect after surgery. The VERION Image-Guided System is effective in guiding the surgeon to achieve accurate CCI during cataract surgery. The current study is potentially meaningful in the context of clinical research because all phacoemulsification procedures were performed by one experienced surgeon. The above-described surgical option may be used more frequently in cataract surgery.

## Methods

### Patients

This prospective study included 212 eyes from 200 patients who underwent phacoemulsification. A single, experienced surgeon (C. K. J.) performed all the procedures at the Seoul St. Mary’s Hospital Eye Center between January 2016 and August 2017. Informed consent was obtained from all of the patients before the commencement of the study. The protocol adhered to the tenets of the Declaration of Helsinki, and St. Mary’s Hospital’s institutional ethics committee approved the study.

All patients underwent a routine comprehensive ophthalmic examination preoperatively. Refractive power and corneal curvature were measured using an auto-keratometer (KR-1, Topcon Medical Systems Inc, New Jersey, USA). Ophthalmoscopy was performed to evaluate the fundus under a dilated pupil. Cataract nuclear density was graded according to the Lens Opacities Classification System III^43^. TCRP, Sim-K and its steep meridian were measured using the Scheimpflug analysis system, Pentacam HR (Oculus Optikgeräte GmbH, Wetzlar, Germany). To ensure sufficient quality, only measurements assigned the quality specification “OK” were accepted as valid. The site of the CCI just in the quadrant of the steep meridian was determined according to patients’ TCRP_4.0_ steep meridian axes.

The inclusion criteria were a diagnosis of cataract with regular corneal astigmatism ranging from 0.50–3.50 D (as determined via an auto-keratometer), a postoperative follow-up period of at least 2 months, a corneal endothelial cell count >2000 per mm^2^, and no viral or bacterial infection. Exclusion criteria were a history of previous ocular surgery, surgical complications, or any corneal or macular pathology, and poor fixation. Cases involving severe dry eye were also excluded.

### Surgical technique

**Figure 3:**
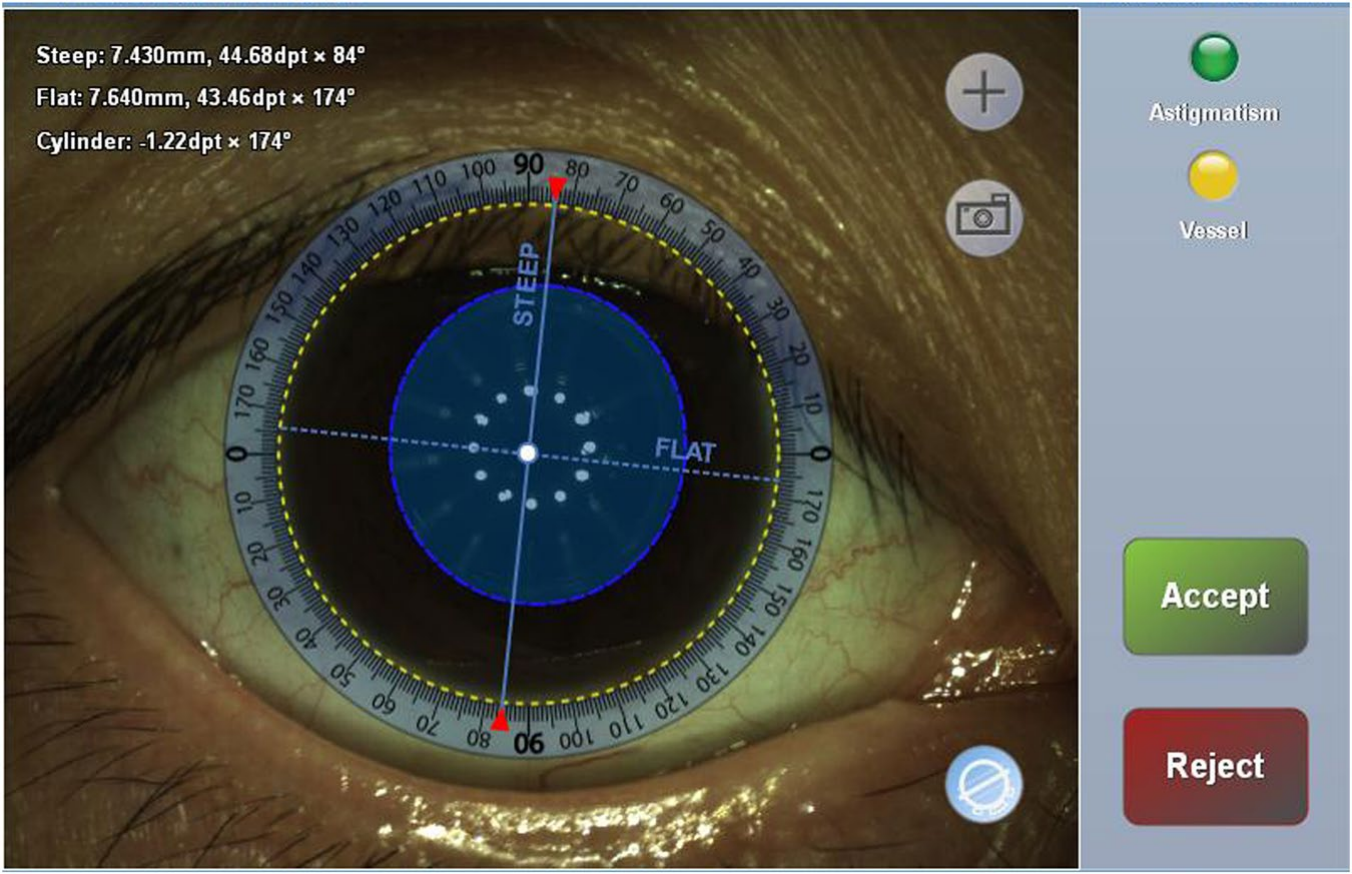
The VERION Image-Guided System was used to determine the steep meridian axis orientation and digital markers for the microscopy.

All phacoemulsification procedures were performed under topical anesthesia (proparacaine 0.5%). The VERION Image-Guided System (Alcon Laboratories Inc., Fort Worth, Texas, USA) was used to determine the steep meridian axis orientation and digital markers for the microscopy (Fig. 3). A 2.2-mm, single-step CCI was made in the pre-limbal region of the steep meridian using a dual-beveled, angled, slit knife (Alcon Laboratories Inc.), and a side port incision (full-thickness puncture) was made using a 1.0-mm knife, 60 degrees left of the main incision site. Lastly, the CCI size was enlarged from 2.3 mm to 3.2 mm for correcting corneal astigmatism and intraocular lens (IOL) insertion. The corneal wounds were left unsutured (sutureless). Preoperative and 2-month postoperative keratometric data were compared. Polar value analysis was used to assess SIA_Cornea_. Patients were divided into four groups based on the incision site: temporal, superotemporal, superonasal (opposite site of incision: inferiortemporal), and superior^4^.

### Methods of calculation

The formula for calculating SIA_Cornea_^19^ is the same as that used for calculating prediction error by Holladay *et al*.^44^. In general, the goal of refractive surgery is to neutralize the refractive error such that the final postoperative refraction is plano. When this is achieved, the Predicted SIA_Cornea_ and the actual SIA_Cornea_ are equal. When the actual SIA_Cornea_ does not match the Predicted SIA_Cornea_, a postoperative refractive error is induced. In cataract surgery with CCI, the intended outcome is postoperative astigmatism of 0.50 D. The differences between preoperative and postoperative polar values were calculated and compared. For example, the preoperative and postoperative astigmatic polar values (AKP) for the preoperative net cylinder A@a and the postoperative net cylinder B@b were defined as follows^19^:

$$\begin{array}{c}\,{\rm{AKP}}{(+0)}_{{\rm{preop}}}={\rm{A}}\\ \,{\rm{AKP}}{(+45)}_{{\rm{preop}}}=0\\ {\rm{AKP}}{(+0)}_{{\rm{postop}}}={\rm{B}}\times \{{\sin }^{2}[({\rm{b}}+90)-{\rm{a}}]-{\cos }^{2}[({\rm{b}}+90)-{\rm{a}}]\}\\ {\rm{AKP}}{(+45)}_{{\rm{postop}}}={\rm{B}}\times \{{\sin }^{2}[({\rm{b}}+45)-{\rm{a}}]-{\cos }^{2}[({\rm{b}}+45)-{\rm{a}}]\}\\ \,\Delta {\rm{AKP}}(\,+\,0)={\rm{AKP}}{(+0)}_{{\rm{postop}}}-{\rm{AKP}}{(+0)}_{{\rm{preop}}}\\ \,\Delta {\rm{AKP}}(+45)={\rm{AKP}}{(+45)}_{{\rm{postop}}}-{\rm{AKP}}{(+45)}_{{\rm{preop}}}\end{array}$$

### Statistical analysis

Descriptive and theoretical analyses were performed using SPSS for Windows, version 18.0 (SPSS Inc, Chicago IL, USA). Data normality was confirmed using the Shapiro-Wilk test. The Wilcoxon Rank-Sum test was used for nonparametric analyses. Comparative evaluation of preoperative and 2-month postoperative keratometric parameters was performed. The Kruskal-Wallis H test was used for multiple comparisons among the four groups. Scatterplots, the R^2^ correlation coefficient, and correlation coefficients (Pearson or Spearman, depending on the data; i.e., whether normality could be assumed) were used to assess the associations between pairs of variables. Hotelling trace test was used for comparison of intraindividual changes, and univariate analysis was used to examine changes in respective polar values in each group. P values < 0.05 were considered statistically significant.
